# Von Hippel-Lindau Disease: A New Approach to an Old Problem

**DOI:** 10.5812/ijem.4510

**Published:** 2012-09-30

**Authors:** Ali Tootee, Shirin Hasani- Ranjbar

**Affiliations:** 1 Tehran University of Medical Sciences, Endocrinology and Metabolism Research Institute, Shariati Hospital, Tehran, IR Iran

**Keywords:** Von Hippel-Lindau Disease, Pheochromocytoma

## Abstract

**Background:**

Von Hippel-Lindau (VHL) disease is a hereditary, autosomal dominant syndrome which is manifested by a range of different benign and malignant tumors. This disease can present with different clinical presentations such as; retinal angioma (RA), hemangioblastoma (HB) of the central nervous system (CNS), pheochromocytoma (Pheo), and epididymal cystadenoma. Tumors are usually accompanied with cysts.

**Objectives:**

As the disease can display different clinical presentations, which are mainly unspecific, and considering the importance of an early diagnosis and the proper and early management of it, this study was carried out to present a general overview of VHL. Moreover, the present article reviews screening methods and emphasizes the need for increasing the awareness of different health care professionals to diagnose and refer the patients in the early stages.

**Materials and Methods:**

A thorough search of internet medical databases, such as PubMed, was carried out on known or suggested; clinical presentations, pathogenesis, screening, causes and criteria for diagnosis of patients and their referrals.

**Results:**

Our research demonstrated that VHL is caused by a mutation in the von Hippel-Lindau (VHL) gene. It also showed that different screening methods can be utilized for the early diagnosis and referral of patients. Different clinical presentations of the disease are also elaborated in some detail and their treatment options are discussed.

**Conclusions:**

Considering the need for a multidisciplinary approach to VHL, especially, given the number of cases which have been reported and diagnosed in Iran, it is of great importance that clinicians remain vigilant in order to identify cases that present with clinical characteristics of the disease, and that they are prompt in referring them to a multidisciplinary VHL clinic. It is also important to establish links with existing VHL Family Alliances and other related organizations around the world.

## 1. Introduction

Von Hippel-Lindau (VHL) disease is a hereditary, autosomal dominant syndrome which is manifested by different benign and malignant tumors (1). A constellation of clinical presentations of VHL disease have been reported including; retinal angioma (RA), hemangioblastoma (HB) of the central nervous system (CNS), pheochromocytoma (Pheo), and epididymal cystadenoma (2, 3).Cysts are usually found around the tumors (4). As VHL is associated with different benign and malignant tumors, which cause high rates of morbidity and mortality, screening and follow up of these patients must be considered to be of paramount importance (1).

## 2. Objective

In this review article, we aim to summarize the recent findings on the molecular pathogenesis, classification, and criteria of clinical diagnosis as well as treatment of VHL disease. Moreover, as a few cases of the disease have been reported in Iran, and considering the fact that currently the Endocrinology and Metabolism Research Institute (EMRI), Tehran, Iran, is prepared to actively participate in early referral of patients and screening of their families for mutations, this review article will be of immense importance to Iranian clinicians for early diagnosis and referral of cases.

## 3. Classifications

Based on clinical manifestations, patients with VHL are classified into two different types: those without pheochromocytoma (type 1) and those with it (type 2).The latter category (type 2) is further divided into three subcategories; type 2A, type 2B and type 2C. VHL type 2Aincludes pheochromocytoma with other HB in the CNS, but without RCC. Type 2B presents with pheochromocytoma, RCC and other CNS tumors. The current opinion is that type 2C disease presents only with Pheo, without other manifestations of the disease. It is noteworthy that hitherto, only a few mutations for VHL type 2C have been identified (3, 5, 6).

A very rare type of the disease is called Chuvash polycythemia. This type, which is caused by VHL gene inactivation at a specific point of the VHL protein, does not result in a tumor, it does, however, lead to polycythemia (3, 7).

## 4. History and Epidemiology

VHL disease was first described in von Hippel’s literature in 1911 and Lindau’s writings in 1926. Subsequently, Melmon and Rosen elaborated the concept of the VHL disease in more detail in 1964 (3). Latif et al. performed positional cloning for the disease with the accumulation of DNA in VHL families, and the VHL gene was identified in 1993. This gene was named as the ‘VHL tumor suppressor gene’, and its location was demonstrated to be on the chromosome 3p25–26. It was later observed that the VHL gene is also inactivated in sporadic renal cell carcinoma, hemangioblastoma and pheochromocytoma (3, 5, 8).

There are two different clinical patterns for diagnosis of the disease: 1) patients with a positive history of developing hemangioblastoma in the; CNS or retinal angioma, renal cell carcinoma, pheochromocytoma, pancreatic tumors or cysts, epididymal cystadenoma, and 2) patients without a family history of VHL who present with hemangioblastoma or retinal angioma in combination with other tumors such as; renal cell carcinoma, pheochromocytoma, pancreatic tumors or cysts, or epididymal cystadenoma (3).

## 5. Molecular Basis

The suppressor gene of the VHL tumor consists of 3 exons and 639 NT (N-terminal). The mRNA of the gene sizes 4.5 kb which has been shown to encode 213 amino acids. Two starting codons exist at the first exon in the VHL gene. It is known that the gene is widely conserved in organisms ranging from Caenorhabditiselegans to human beings. The development of VHL-associated disorders has been elaborated with the use of the classic theory of Knudson’s two-hit hypothesis, which proposes that both VHL alleles are inactivated as a result of mutation and deletion of the VHL gene, which in turn, results in functional impediment of the tumor suppressor gene. A recent functional study of the VHL gene has shown that mRNA is transcribed from both starting codons (9-11).

Various tumors, such as HB in the CNS, RA, Pheo, RCC, neuroendocrine tumors in the pancreas, renal, and pancreatic cysts, have been found to develop through the same process as that in the inactivation of the VHL gene and subsequent functional loss of the pVHL-elong in B-C (VBC) complex. It has been demonstrated that the VHL protein has an a- or b-domain either in the 30 section or in the middle portion of this gene. The a-domain is functionally important for binding other proteins such as Elongin BC. The function of the b-domain is to facilitate binding target ubiquitinated proteins for the VBC complex. The VBC complex acts as the E3 ligase for ubiquitination and further proteasomal degradation of target proteins, therefore, any damage to functional VHL protein can lead to the accumulation of target proteins, eg, HIF1a, HIF2a as well as atypical protein kinase l. HIFs have been proven to act as transcription factors in hypoxic situations. The b-domain of the VHL protein of the Elongin BC complex (VBC) is the site that they usually bind to (3). Under normal oxygen pressure, however, they are usually believed to be ubiquitinated and degraded by the VBC. In contrast, they are not degraded in a hypoxic situation. As this mechanism is hauled with any defect in the function of the VHL protein, a high level of non-degraded HIF causes increased transcription of VEGF, PDGF and TGF-a (5, 12-14).

These findings shed some light on the growth of the cell and the proliferation of micro vascular structures, as well as the increased rate of growth in HB, RCC or other VHL-related tumors. HIF leads to an increase in the production of tyrosine hydroxylase, and subsequently, overproduction of catecholamine in VHL-related pheochromocytoma. It has been demonstrated that any defect in the function of the VBC complex also results in the accumulation of atypical protein kinase l. Moreover, it can increase the production of inhibitors of neural crest cells apoptosis in the medullary tissue of the adrenal gland This is considered to be one of the main causes of the development of pheochromocytoma (5, 12-14).

## 6. Pathogenesis 

It has been demonstrated that VHL disease originates from mutations of the von Hippel-Lindau (VHL) gene. This familial autosomal-dominant syndrome can lead to the development of a number of benign and malignant tumors. Those tumors include; CNS and retinal hemangioblastomas, pheochromocytomas, and clear cell renal carcinomas. At least 30% of the disease-causing mutations in the VHL gene need to be involved in order to cause clinical manifestations. Identification of these mutations is not possible using PCR-based mutational scanning methods. Traditionally, quantitative Southern blot analysis has been utilized for the detection of complete or partial deletions and further alternations in the gene (15).

## 7. Screening

### 7.1. Genetic Evaluation

Different studies have demonstrated that the early onset age of RCC is a common feature of the disease. It usually affects people in their third, fourth or fifth decade of life and kidney involvement is usually bilateral and multifocal. The results of several studies have suggested that deletions could play a role in increasing the risk of RCC development in families with a positive VHL history. Since the life expectancy of patients with VHL mutations is less than 50 years, the prognosis may be improved by an early screening and diagnosis. Therefore, patients and their offspring should be screened for VHL mutations by means of molecular genetic testing. Early detection of affected gene carriers has been suggested as a key role in early diagnosis and improved prognosis (9).

### 7.2. Ophthalmology Screening

In one study, Hasani-Ranjbar et al. suggested that as visual loss was one of the major complications of VHL disease, ophthalmologic screening of offspring and relatives of patients with the disease, could play a key role in screening for the disease (1).

### 7.3. Audiometry

Annual audiometry could play a role as a first-line endolymphatic sac tumor screening tool. It is also recommended that in countries where periodic surveillance magnetic resonance imaging of the central nervous system is available, specific images of the inner ear should be recorded. Audiometric signs in patients suffering from VHL disease, who do not present with magnetic resonance imaging-visible endolymphatic sac tumors, can be related to microscopic endolymphatic sac tumors. It has been shown that the study of audiometric endolymphatic sac tumor characteristics plays a key role in screening, and this in turn, can improve endolymphatic sac tumor prognosis (11). 

### 7.4. Screening for Pheochromocytoma

As pheochromocytoma can be the first clinical presentation of VHL disease, it seems feasible to screen for it in order to diagnose VHL cases. There are different plasma or urine tests for screening of the disease, however, the preferred biochemical screening test is usually measurement of fractionated metadrenalines such as methoxytyramine (16).

## 8. Clinical Manifestations, Diagnosis, Treatment, and Prognosis 

VHL disease is a multi systemic disorder which predisposes the affected individuals to a wide range of benign and malignant tumors and cysts.

### 8.1. Hemangioblastoma in the CNS

CNS presentations are common in VHL patients and CNS tumors are the leading cause of a great proportion of morbidity and mortality in these patients. CNS neoplasms in patients with VHL usually develop below the tentorium. They usually occur in the cerebellum, brainstem, and spinal cord. A few cases of hemangioblastoma have been reported to be located in the caudaequina (17).

As most patients with VHL show presentations of craniospinal hemangioblastomas, it is recommended that optimal management of these neoplasms is vital, in order to minimize morbidity and mortality (8, 18, 19).

### 8.2. Retinal Hemangioblastoma

The onset of the development of retinal hemangioblastoma can range from less than 10 years of age until the age of 30 years, an age similar to that of the development of HB in the CNS. After the age of 30, the risk of RA development gradually decreases. It afflicts patients with both VHL type 1 and VHL type 2 diseases. Usually only one tumor develops in one eye and there are no symptoms associated with the disease (3). As mentioned previously, Hasani-Ranjbar et al. have suggested a diagnostic role for ophthalmology in the early screening and diagnosis of disease in the relatives of those diagnosed with the disease (1).

### 8.3. Pheochromocytoma

Pheochromocytomas (PHEOs) and paragangliomas (PGLs) are catecholamine-releasing tumors which originate from chromaffin cells of the adrenal medulla and extra-adrenal sites. Extra-adrenal located pheochromocytomas are known as paragangliomas. The prevalence of PHEOs and PGLs are 1:4500 and 1:1700 respectively, with an overall annual incidence of 3-8 cases per 1 million per year in the general population has been reported (20).

Malignant forms of catecholamine-secreting tumors are relatively rare and the rate of malignancy is reported to be 2.4-2.6%. It is noteworthy, that there is currently no histological evidence of malignancy for such tumors and the only suggested criterion of malignancy is the presence of metastasis (1). The distant metastases are usually of hematologic origins, and they mainly affect; bone, liver and lung tissues (20). The prevalence of metastasis is estimated to be up to 36-50% for extra-adrenal abdominal pheochromocytoma and 10% and 5% for adrenal and familial types respectively (20).

Most pheochromocytomas appear in the form of sporadic tumors. However, in some cases, the disorder presents as part of a familial disorder (15-30%). Sporadic pheochromocytomas usually present as unicentric and unilateral legions, while familial ones are often metacentric and bilateral. Hereditary pheochromocytoma typically present at a younger age compared to the sporadic forms of the disease (20). Moreover, some familial disorders present with pheochromocytoma such as; VHL syndrome, multiple endocrine neoplasia type 2 (MEN2), neurofibromatosis type 1, and SDH mutation-related tumors (20).

Pheochromocytoma develops in paraganglia or the adrenal glands in type 2 VHL disease, which comprises some 10% of VHL disease cases. Some studies have suggested that its development can occur in different age groups, from < 10 years to > 40 years (3). Specific point mutations in the VHL gene have been demonstrated, some of which are detected at exon 3 at high frequencies. They are usually located at or near the binding region of Elongin C. 

Several diagnostic tests are employed for the diagnosis of pheochromocytoma. The most widely used test is evaluation of 24 hour urine for catecholamine metabolites. In regard to metastatic lesions; abdominal computed tomography (CT), magnetic resonance imaging (MRI), and 131iodine-metaiodobenzylguanidine (MIBG) are utilized.

The treatment for pheochromocytoma is surgical removal of the lesions. Since it is possible to diagnose the small size of these lesions prior to the initiation of major symptoms, depending on the medical technique employed, they may be excised by a laparoscopic technique with very low morbidity (3). Partial adrenalectomy is usually preferred in patients with VHL syndrome with pheochromocytoma, and the outcomes are encouraging at long-term follow-up. Adrenal sparing surgery, can result in obviating the need for steroid replacement, in many patients (21).

### 8.4. Renal Cell Carcinoma and Renal Cysts

VHL disease, being a multisystemic disorder, predisposes patients to renal cysts and cancer. Moreover, one study has shown that fast tumor growth increases the risk of metastases (22). However, the majority of renal masses with VHL disease are reported to be indolent and these are not likely to metastasize during long-term follow-up, even in tumors larger than 3 cm. Generally, metastatic potential during active surveillance is observed to be low even in VHL patients with renal tumors > 4 cm (23).

As discussed earlier, the VHL gene protein has different functions which are aimed at the suppression of tumors. The best known and directly proven factor linked with the development of renal cell carcinoma (RCC) is the inhibition of hypoxia-inducible factor (HIF). This inhibition has also been shown to play a role in targeting HIF for ubiquitin-mediated degradation (24). HIF plays a critical role in pVHL-defective tumor formation, and it has also been suggested that HIF antagonists or its downstream targets (such as vascular endothelial growth factor), may have a potential role in the treatment of RCC in the future. Currently, several drugs have been developed that target HIF-responsive gene products, and many of these targeted therapies have proved to be effective in kidney cancer clinical trials thus heralding a new era in RCC therapy (24).

### 8.5. Pancreatic Lesions

Although VHL disease usually presents with; hemangioblastoma of the central nervous system and retina, clear cell renal cell carcinoma, and pheochromocytoma, it is well known that other organs can be affected as well. Pancreatic lesions, both primary and metastatic, are commonly detected, therefore, they must be considered by clinicians when a diagnosis of VHL is made (25).

## 9. Prognosis, Postoperative Complications, and Recent Antiangiogenic Therapy

The prognosis of VHL disease was once considered to be related to the outcome of RCC treatment. RCC is the most serious tumor, with high metastasizing potential to other organs in VHL disease. However, with the recent emergence of novel surgical techniques such as, partial adrenalectomy and nephron-sparing surgery, as well as non-surgical approaches such as, radiofrequency ablation treatment, VHL has become a curable condition. A considerable risk of morbidity is reported to originate from postoperative complications caused by the treatment of (spinal cord) HB. Such complications usually consist of a wide range of neurologic damage including; paraplegia, and sensory and motor disturbances (3).

Recently, new drugs have been developed and their therapeutic effects are being investigated by means of clinical trials. These medications include anti-angiogenic agents which are used in the treatment of RCC. Although thalidomide with interferon, or SU5416 alone was demonstrated to halt tumor growth in RCC, it did not reduce the size of it. There are also other molecular targeting drugs designed to act as inhibitors of VEGF receptor kinase, which are in the process of clinical trials. These drugs have proved to be very promising in terms of their clinical effects, demonstrating a partial response in some 40-90% of cases as well as stabilization of the disease. Moreover, some inhibitors designed specifically for HIFs may prove to be effective in the future (3, 26, 27).

## 10. Conclusions

It can be asserted that familial support groups for VHL need to be integrated into healthcare systems worldwide, taking in to consideration the above mentioned characteristics of the disease. Such groups for VHL disease are increasingly being organized in different countries and they provide the opportunity for mutual cooperation for both the VHL patients and their families. VHL Family Alliances are active in the US, Canada, England, France, Germany, Italy and Japan (25). Unfortunately, no such organization currently exists in Iran.

It can be argued that it is of the utmost importance for Middle Eastern healthcare providers and governments to found and organize well equipped centers for the referral and management of patients with different manifestations of the disease. It is noteworthy, that one such referral center is currently being established at the Endocrinology and Metabolism Research Institute (EMRI) in the Tehran University of Medical Sciences (TUMS), and genetic testing for suspicious cases is currently available there.

Obviously, there is the need for a clear set of criteria for the referral of suspected cases and the criteria should be based on different countries’ resources and limitations. The reference criteria for the referral of suspicious cases to the VHL clinic, Massachusetts General Hospital (Boston, USA), is tabulated in Table to demonstrate that a similar set of criteria could be proposed as the criteria for genetic evaluation (28).

**Table:** Criteria for Referral to Massachusetts General Hospital VHL Clinic (28)

Any Blood Relative of an Individual Diagnosed With VHL Disease	
Any individual with TWO VHL-associated lesions	
	Hemangioblastoma
	Clear cell renal carcinoma
	Pheochromocytoma
	Endolymphatic sac tumor
	Epididymal or adnexal papillary cystadenoma
	Pancreatic serous cystadenomas
	Pancreatic neuroendocrine tumors
Any individual with ONE or more of the following	
	CNS hemangioblastoma
	Pheochromocytoma or paraganglioma
	Endolymphatic sac tumor
	Epididymal papillary cystadenoma
Any individual with	
	Clear cell renal carcinoma diagnosed at age < 40 years
	Bilateral and/or multiple clear cell RCCs
	> 1 Pancreatic serous cystadenoma
	> 1 Pancreatic neuroendocrine tumor
	Multiple pancreatic cysts + any VHL associated lesion

As treatment and follow-up of patients is a long-term process and many require health services for a long period of time, possibly their whole lives, the VHL center needs to be well equipped and trained healthcare providers from a wide range of specialties should be employed. The center needs to employ; surgeons, neurosurgeons, endocrinologists, genetic counselors, and psychologists, who are experts in this specialized field.
